# Supplementary material for: Desiccant Films Made of Low-Density Polyethylene with Dispersed Silica Gel—Water Vapor Absorption, Permeability (H2O, N2, O2, CO2), and Mechanical Properties
Source: Materials (Basel). 2019 Jul 18;12(14):2304. doi: 10.3390/ma12142304 (PMC6679128; doi:10.3390/ma12142304)
Supplement: Supplementary file 1 [file materials-12-02304-s001.pdf]

materials

Article

Desiccant Films Made of Low-Density Polyethylene with Dispersed Silica Gel—Water Vapor Absorption, Permeability (H_2O, N_2, O_2, CO_2), and Mechanical Properties

Sven Sängerlaub *, Esra Kucukpinar and Kajetan Müller

The volume and therefore the length of the films with dispersed silica gel increased with time when exposed to 91% RH, 23 °C, measured after 260 days for each of the samples as shown in following Figure. The elongation is dependent on the silica powder concentration. Reasons can be the relaxation of the polymers or the volume increase of the silica gel by water vapour absorption.

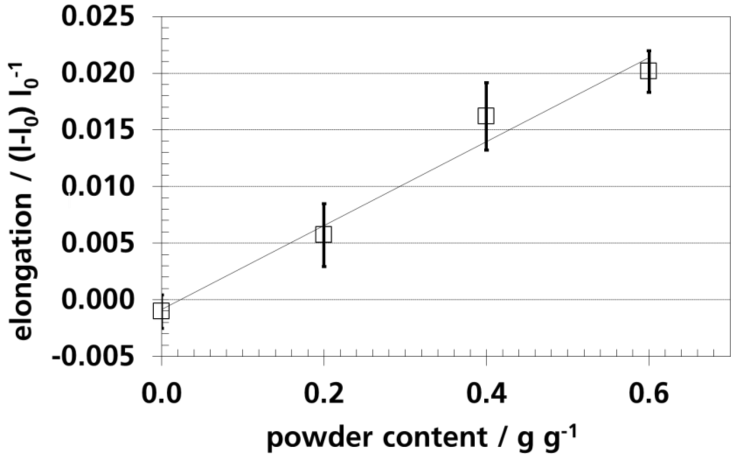

Figure S1. Elongation by water vapour absorption. Elongation of PE-LD samples with different amounts of silica gel -powder after 260 days; samples stored at 91% RH and 23 °C.

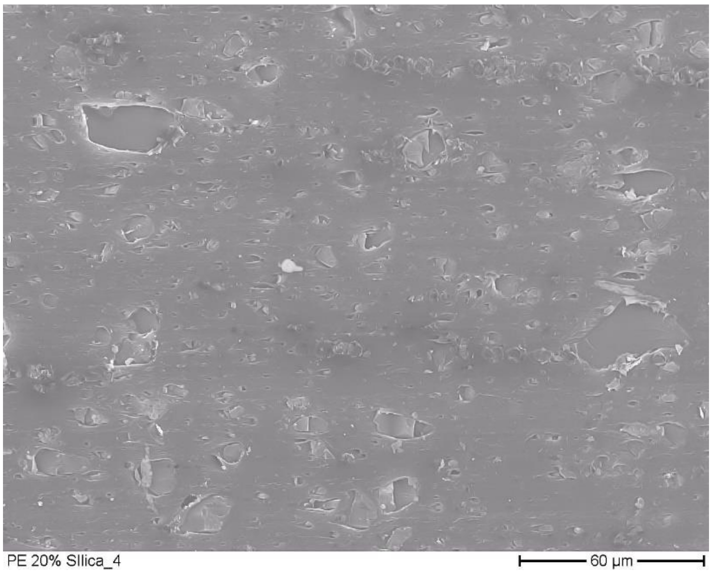

materials
MDPI

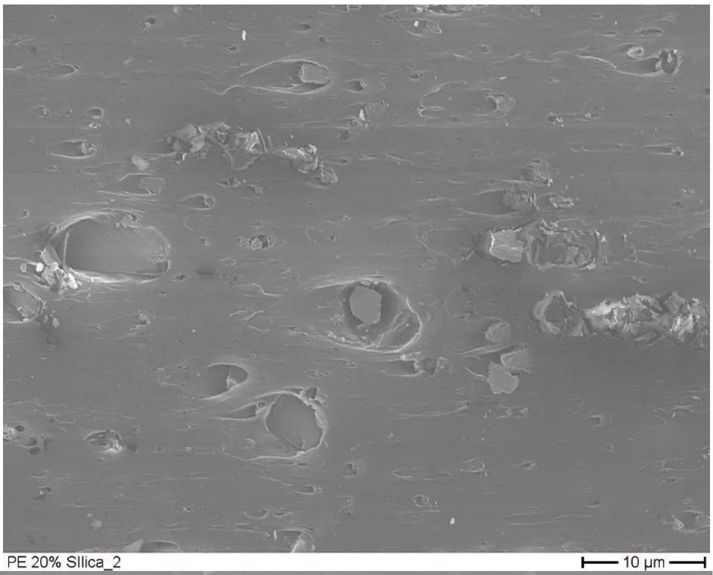

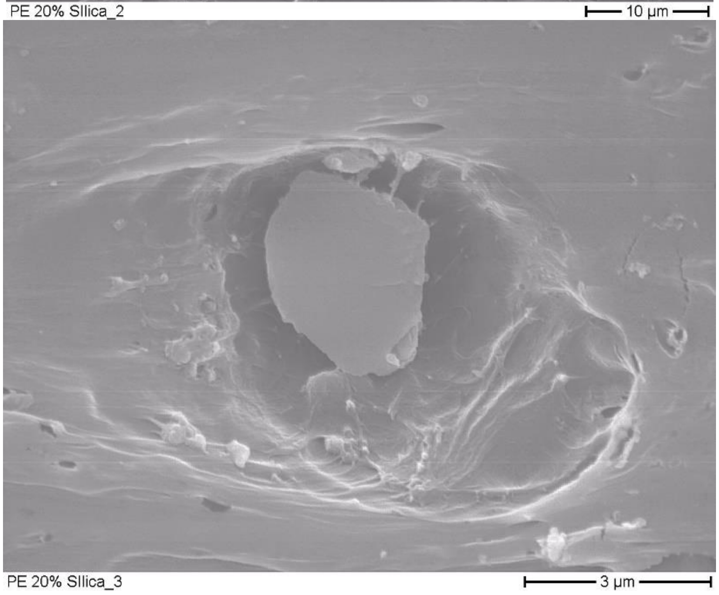

(a)

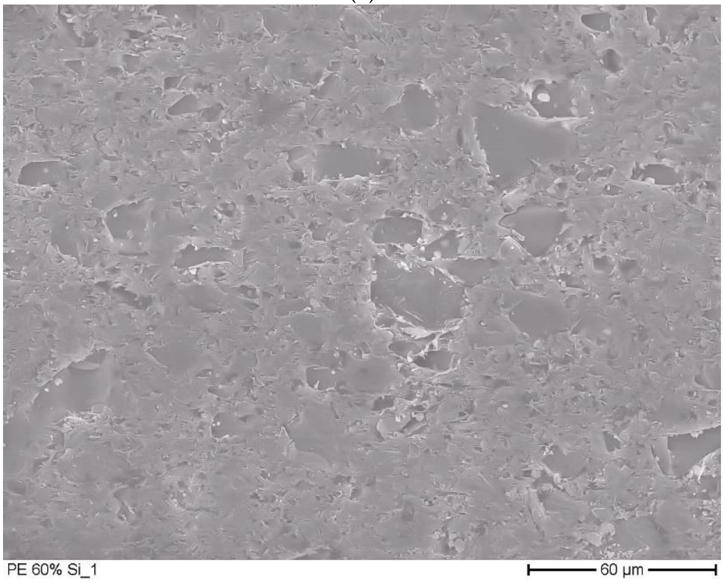

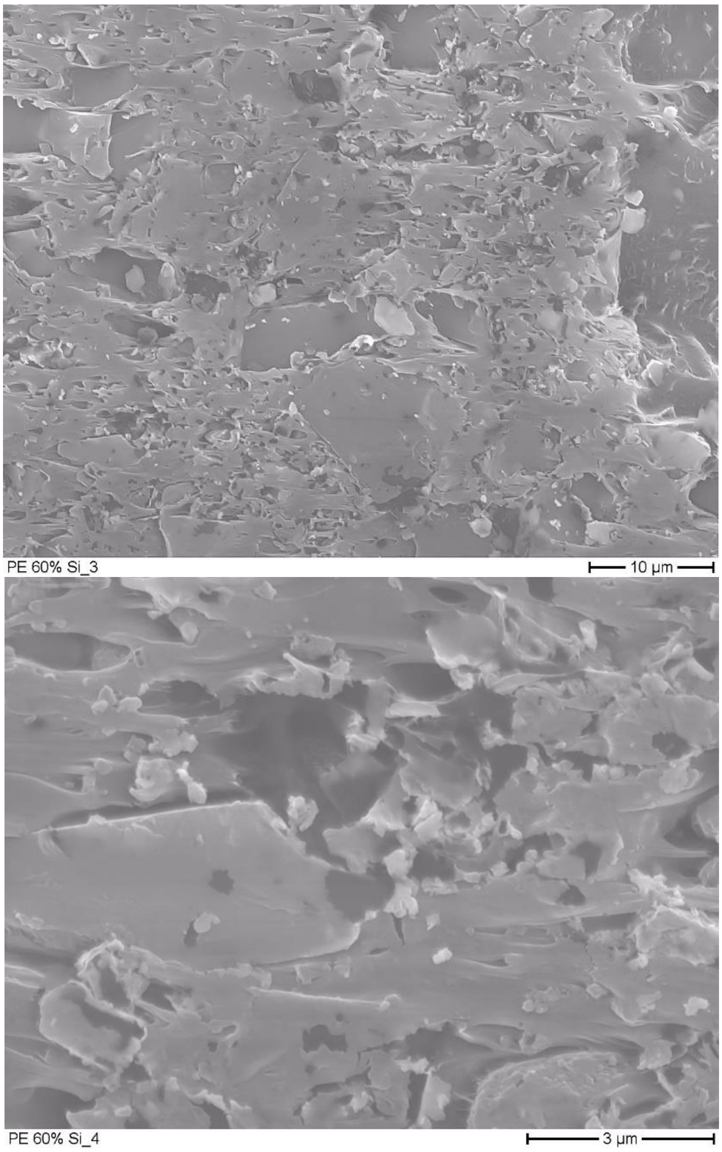

(**b**)

Figure S2. Electron microscopic pictures of the sample films in different magnifications. (**a**) Electron microscopic pictures of film with 0.2 g silica gel/g film; (**b**) Electron microscopic pictures of film with 0.6 g silica gel/g film.

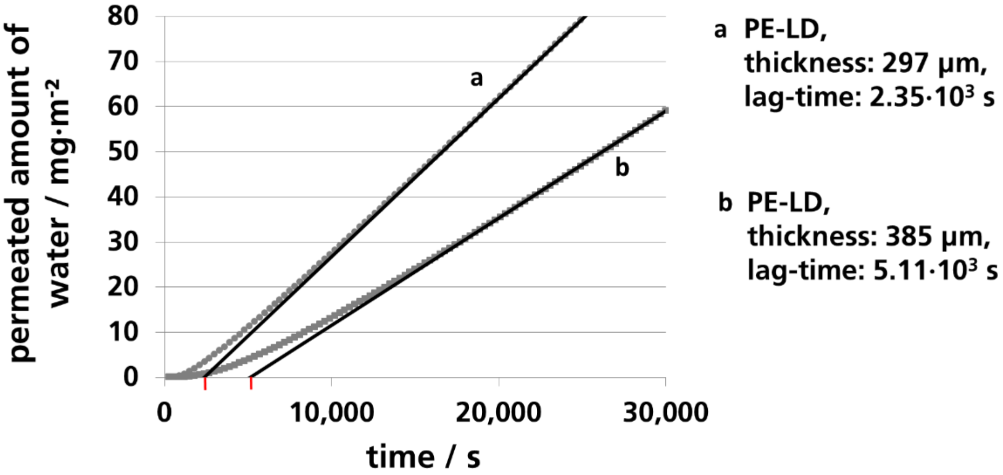

(**a**)

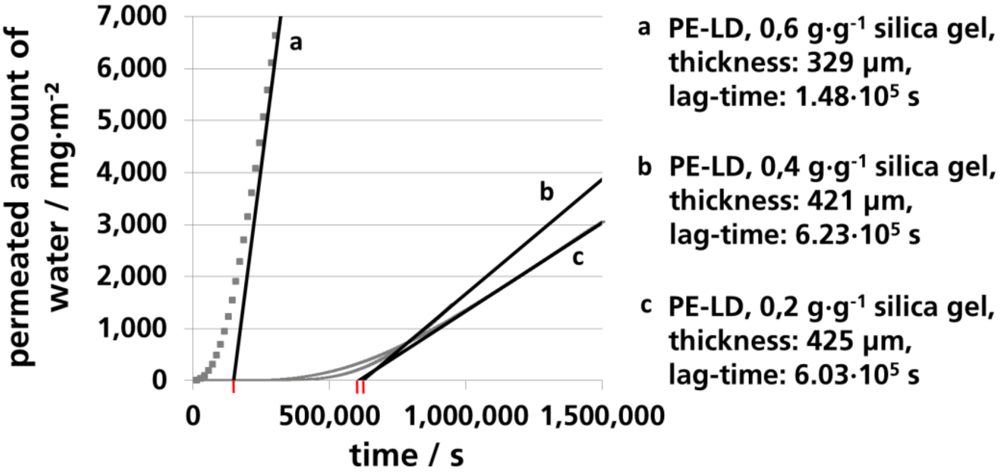

(**b**)

Figure S3. Determination of (effective) diffusion coefficients by lag-time measurements; (**a**) Determination of lag-times of PE-LD films; 23 °C; (**b**) Determination of lag-times of PE-LD films with dispersed silica gel; 23 °C.
